# Assessing Marine Microbial Induced Corrosion at Santa Catalina Island, California

**DOI:** 10.3389/fmicb.2016.01679

**Published:** 2016-10-25

**Authors:** Gustavo A. Ramírez, Colleen L. Hoffman, Michael D. Lee, Ryan A. Lesniewski, Roman A. Barco, Arkadiy Garber, Brandy M. Toner, Charles G. Wheat, Katrina J. Edwards, Beth N. Orcutt

**Affiliations:** ^1^Department of Biological Sciences, University of Southern California, Los AngelesCA, USA; ^2^Department of Earth Science, University of Minnesota-Twin Cities, MinneapolisMN, USA; ^3^Department of Soil, Water, and Climate, University of Minnesota-Twin Cities, St. PaulMN, USA; ^4^Global Undersea Research Unit, University of Alaska Fairbanks, Moss LandingCA, USA; ^5^Bigelow Laboratory for Ocean Sciences, East BoothbayME, USA

**Keywords:** microbial induced corrosion (MIC), mineral–microbe interactions, accelerated low-water corrosion (ALWC), Catalina Island, Wrigley Institute

## Abstract

High iron and eutrophic conditions are reported as environmental factors leading to accelerated low-water corrosion, an enhanced form of near-shore microbial induced corrosion. To explore this hypothesis, we deployed flow-through colonization systems in laboratory-based aquarium tanks under a continuous flow of surface seawater from Santa Catalina Island, CA, USA, for periods of 2 and 6 months. Substrates consisted of mild steel – a major constituent of maritime infrastructure – and the naturally occurring iron sulfide mineral pyrite. Four conditions were tested: free-venting “high-flux” conditions; a “stagnant” condition; an “active” flow-through condition with seawater slowly pumped over the substrates; and an “enrichment” condition where the slow pumping of seawater was supplemented with nutrient rich medium. Electron microscopy analyses of the 2-month high flux incubations document coating of substrates with “twisted stalks,” resembling iron oxyhydroxide bioprecipitates made by marine neutrophilic Fe-oxidizing bacteria (FeOB). Six-month incubations exhibit increased biofilm and substrate corrosion in the active flow and nutrient enriched conditions relative to the stagnant condition. A scarcity of twisted stalks was observed for all 6 month slow-flow conditions compared to the high-flux condition, which may be attributable to oxygen concentrations in the slow-flux conditions being prohibitively low for sustained growth of stalk-producing bacteria. All substrates developed microbial communities reflective of the original seawater input, as based on 16S rRNA gene sequencing. Deltaproteobacteria sequences increased in relative abundance in the active flow and nutrient enrichment conditions, whereas Gammaproteobacteria sequences were relatively more abundant in the stagnant condition. These results indicate that (i) high-flux incubations with higher oxygen availability favor the development of biofilms with twisted stalks resembling those of marine neutrophilic FeOB and (ii) long-term nutrient stimulation results in substrate corrosion and biofilms with different bacterial community composition and structure relative to stagnant and non-nutritionally enhanced incubations. Similar microbial succession scenarios, involving increases in nutritional input leading to the proliferation of anaerobic iron and sulfur-cycling guilds, may occur at the nearby Port of Los Angeles and cause potential damage to maritime port infrastructure.

## Introduction

**FIGURE 1:**
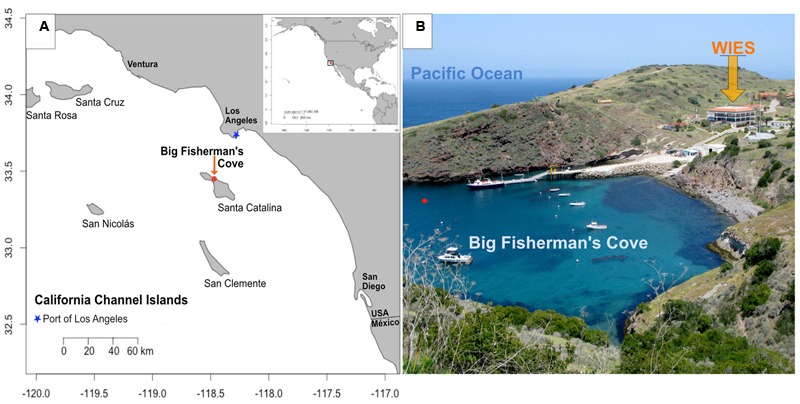
**Location of study site. (A)** Map of Santa Catalina Island, CA, USA, a California channel island near the Port of Los Angeles, with latitude and longitude shown on y- and x-axes, respectively; **(B)** Experiments were conducted in flowing seawater tanks at the Wrigley Institute for Environmental Studies (WIES) sourcing seawater from Big Fisherman’s Cove from the location marked with a red dot in **(B)**. Photo credit: Dieuwertje Kast.

Novel applications of microbial monitoring technologies are of particular importance near the port of Los Angeles (**Figure 1A**), the busiest port in the Western Hemisphere, where complex ecological responses to leaching iron and trophic variability can potentially negatively affect port infrastructure and associated local/global economies (Gubner and Beech, 1999; Beech and Sunner, 2004; Beech and Campbell, 2008). The presence of microorganisms as causative agents of iron infrastructure damage and the financial impact of such processes on society have been documented since the early 20th century (Von Wolzogen Kühr and van der Vlugt, 1934). Recent studies highlight the increased incidence of a biocorrosion phenomenon called accelerated low-water corrosion (ALWC). ALWC is a microbial induced corrosion (MIC) state presumably primed by eutrophic conditions conducive to the rapid recruitment and establishment of iron and sulfur cycling microbial cohorts capable of severe maritime infrastructure damage (Beech and Sunner, 2004; Little et al., 2007, 2013; Beech and Campbell, 2008; Dang et al., 2011).

Marine iron-oxidizing bacteria (FeOB) are oxygen-dependent lithotrophs capable of iron oxyhydroxide (FeOOH) precipitation in circumneutral pH environments (Emerson et al., 2010). FeOB have been previously investigated in deep (Emerson and Moyer, 2002; Edwards et al., 2004) and near-shore (Dang et al., 2011; McBeth et al., 2011; McBeth and Emerson, 2016) marine environments. FeOB have important biogeochemical and oxidation–reduction (redox)-related roles along oxygen gradients in high-Fe environments, leading to Fe^2+^ dissolution rates that are nearly an order of magnitude higher relative to kinetically sluggish abiotic controls (Edwards et al., 2003b, 2004). Additionally, FeOB may further enhance corrosion of mild steel in marine environments by priming substrate surfaces for subsequent colonization of anaerobic microbes in anoxic biofilm microniches, concomitantly increasing Fe^2+^ solubilization (McBeth et al., 2011) and halting advective intrusion of oxygen (Schramm et al., 1999). Anaerobic microbial consortia capable of Fe (III) and sulfate reduction preferably adhere to biogenic rather than synthetic iron oxyhydroxides (Emerson, 2009; Langley et al., 2009). Thus, FeOOH bioprecipitates deposited under oxic, high Fe^2+^, and eutrophic conditions, may spur microbial corrosion of ferruginous maritime infrastructure (Blothe and Roden, 2009; Dang et al., 2011; McBeth et al., 2011; Marty et al., 2014).

**FIGURE 2:**
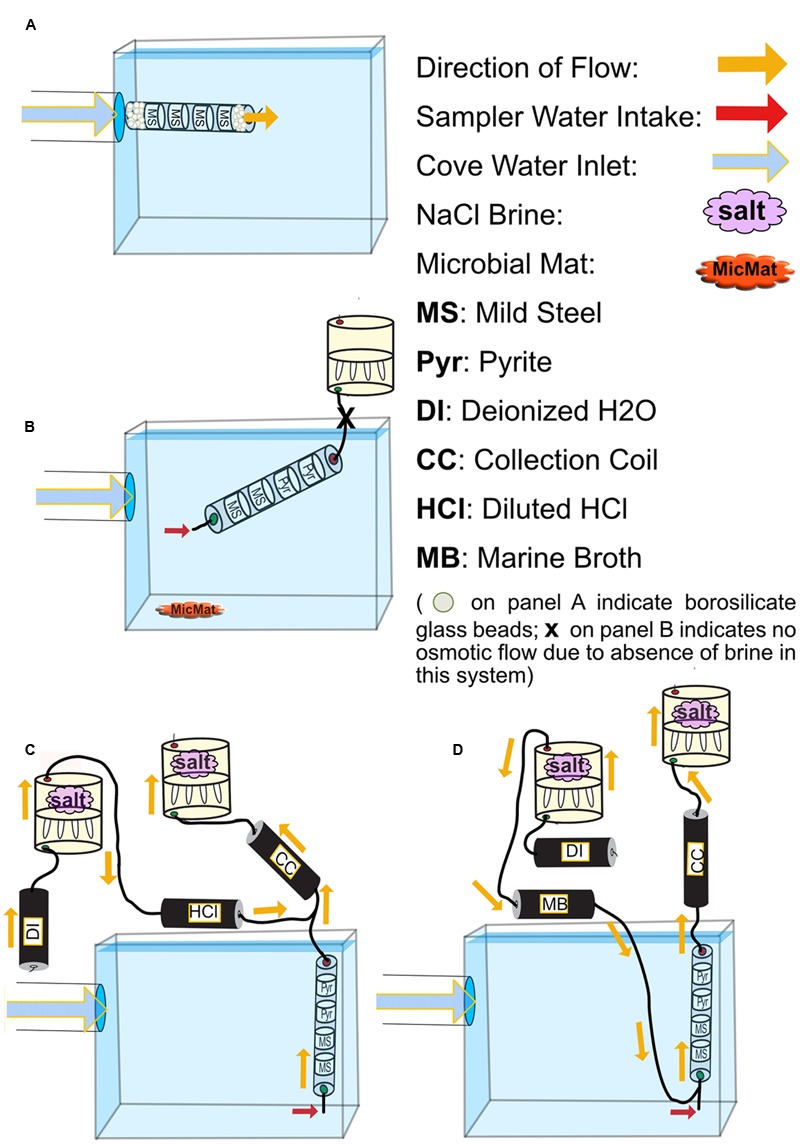
**Schematics for all FLOCS deployment configurations. (A)** high-flux microbial colonization (HFMC): the high-flux colonization system is connected directly to the PVC pipe delivering cove water into the tank. Mild steel shavings are prevented from exiting the cylindrical sleeve due to high flow rates by compacted borosilicate beads. **(B)** SC-FLOCS: stagnant condition configuration where, due to the absence of salt in the osmotic pump, no active intake of environmental sample occurs. Also shown is a representation of microbial mat sample collected from this tank. **(C)** AF-FLOCS: only environmental sample flows through the colonization chamber, and diluted HCl is delivered down stream to preserve redox-sensitive ions in the collection coil for subsequent geochemical analyses. **(D)** NE-FLOCS: nutritional enrichment condition where marine broth and environmental sample are both delivered to colonization chambers.

To explore the local microbial environmental response to high iron and nutritional enrichment in an environment near the Port of Los Angeles, we deployed microbial colonization experiments in laboratory-based aquariums flushed with surface seawater from Big Fisherman’s Cove at Santa Catalina Island (**Figures 1** and **2**; **Table 1**). The colonization experiments introduced surface seawater to mild steel and/or pyrite under various conditions of flow rates and nutrient stimulation, to explore the combined effects of Fe^2+^, nutrients, and oxygen on microbe–substrate interactions, mimicking environmental conditions attributed to ALWC. We hypothesized that microbial community response to iron leaching of ferruginous substrates (mild steel and pyrite) under nutrient stimulation would result in differences in microbial community composition and structure relative to non-nutritionally enhanced conditions. Additionally, we expected nutritionally enhanced substrates to host substrate-attached communities dominated by FeOB, as others have suggested that FeOB play a pivotal role as early colonizers in MIC (Dang et al., 2011; McBeth et al., 2011; McBeth and Emerson, 2016). A combination of electron and X-ray microscopy, high-throughput 16S rRNA gene sequencing of environmental DNA, and geochemical monitoring suggests an oxic-to-anoxic microbial succession scenario in our incubations. We observe an abundance of Delta- and Epsilon-Proteobacteria in slow-flow 6-month colonization experiments coupled with a dearth of biogenic FeOOH precipitates, and abundant black metal sulfides, suggesting that initial biogenic FeOOH deposition by FeOB provides Fe (III) reduction sites for iron reducing bacteria (FRB), as shown elsewhere (Lee et al., 2013). We conclude that iron oxidation by FeOB leads to decreasing oxygen amounts and concomitant growth of microbial communities comprised of facultative and obligate anaerobes whose closest cultured representatives are known for Fe-reduction and S-cycling. As suggested by others, the Zeta- and Epsilon-proteobacteria likely play an important role in the corrosion of ferruginous substrates (Dang et al., 2011; McBeth et al., 2011). We suggest that their involvement may be temporally decoupled and redox-dependent in our experiments. Further, we propose that H_2_S, the metabolic by-product of sulfate reducing bacteria (SRB), may lead to the recruitment of sulfide oxidizing bacteria (SOB) as the final step in a 3-tier (FeOB → SRB/FRB → SOB) ecological recruitment strategy for ferruginous substrate marine microbial colonization, a process likely accelerated under eutrophic conditions with potential ramifications for the enhanced MIC state known as ALWC near a globally important port.

**Table 1:** Summary of samples used in this study.

Sample ID	Description
HFMC	High-flow microbial colonization chamber, mild steel shavings only, connected directly to aquarium inflow pump
SC-FLOCS	Stagnant condition flow-through osmo colonization system (FLOCS), mild steel shavings (MS) and pyrite granules (Pyr), passive flow through intake only with no pump
AF-FLOCS	Active flow-FLOCS, mild steel shavings (MS) and pyrite granules (Pyr), outflow connected to OsmoPump (with eﬄuent fixed with dilute acid)
NE-FLOCS	Nutrient enrichment-FLOCS, mild steel shavings (MS) and pyrite granules (Pyr), outflow connected to OsmoPump and intake mixed with sterile (1:6 dil) LB media
MicMat	Iron oxide microbial mat material from bottom of aquarium at end of experiment
Seawater	Ambient seawater in aquarium at end of the experiment

## Materials and Methods

### Experiment Deployment

To assess microbial mineral colonization in surface seawater under controlled conditions, we used the flow-through osmo colonization system (FLOCS) approach designed for use in the deep sea (Orcutt et al., 2010; Wheat et al., 2011). FLOCS are osmotically driven microbial colonization chambers that allow for long-term monitoring of microbiological and geochemical processes on mineral substrates. FLOCS typically consist of three primary components, in order of the direction of flow: colonization chambers made of plastic sleeves housing mineral substrates, a fluid collection coil, and an osmotic pump (**Figure 2**). In this experiment, colonization chambers consisted of a polycarbonate cylindrical sleeve that contained sterile (autoclaved) mild steel shavings (made by drilling into 0.125″ × 0.25″ 1018 Cold Finish Mild Steel Rectangle Bars)^1^, and/or crushed pyrite (>250 μm size fraction, Ward’s Science, Catalog 466448) in sterile, nutrient deplete seawater from the Sargasso Sea (commercially available from Sigma-Aldrich, S9148-1L).

In this study, a single “free-flow” colonization chamber without attached fluid collection coil or osmotic pumps, and three independent FLOCS containing mild steel and pyrite (FeS_2_) under various flow and nutrient scenarios, were deployed in aquarium tanks at the Wrigley Institute for Environmental Science (WIES) located in Big Fisherman’s Cove at Catalina Island, CA, USA (**Figure 1B**). The glass aquarium tank (189 L) was connected to a PVC piping system that pumps unfiltered seawater from the cove, from ∼10 m water depth, at a rate of 5 L per minute into and out of the tank. The tank volume replacement time is approximately 30 min. To preclude algal growth in the aquarium, the tank was completely covered in black plastic to keep the experiments in darkness. In these experiments, only the colonization chamber was submerged in the aquarium tank, while the Teflon tubing and osmotic pumps for the FLOCS were placed outside of the tank and secured on stands (**Figure 2**).

Four colonization conditions were used in this study (**Figure 2**; **Table 1**):

(1) A high-flux microbial colonization (HFMC) chamber, containing mild steel and 3-mm-diameter glass beads (Fisher Scientific, Catalog No. 10-310-1), was deployed for 2 months in direct connection to the aquarium inflow, with no fluid collection coil or osmotic pump (**Figure 2A**). The HFMC was the only deployment sampled in 2-week intervals for the duration of the 8-week deployment.
(2) A Stagnant Condition (SC-FLOCS) with no osmotic pumping and restricted outflow (**Figure 2B**); therefore “active” osmotic pumping of sample through the colonization chamber did not occur, but diffusive exchange of seawater could occur at the inlet of the chamber.
(3) An Active Flow (AF-FLOCS) with seawater intake driven by an osmotic pump, and with *in situ* acid-preservation of the outflow for geochemical analysis (**Figure 2C**). Similar to the “acid configurations” described elsewhere (Wheat et al., 2010), the AF-FLOCS deployment included a second two-membrane osmotic pump and Teflon coil to deliver 6N HCl to the intake of the collection coil (**Figure 2C**). Acid addition maintains a low pH environment in the Teflon sample coil, keeping dissolved metals in solution.
(4) A Nutrient Enrichment FLOCS (NE-FLOCS) where seawater was mixed with marine nutrient broth and pulled through the colonization chamber via an attached osmotic pump (**Figure 2D**). The NE-FLOCS deployment consisted of a two-membrane osmotic pump delivery system that introduced marine nutrient broth solution (Marine Broth in a 1/6 ratio, used for the cultivation of heterotrophic marine bacteria, BD Difco Product # 279110) into the intake to the colonization chamber (**Figure 2D**), following a concept described as “enrichment” elsewhere (Wheat et al., 2011).

The HFMC experiment was deployed from July 20th to September 20th, 2012. FLOCS experiments were started on July 17, 2012, and terminated January 9, 2013, for a total of 176 days. Daily water temperatures in Fisherman’s Cove typically vary from 12 to 22°C, on average during these seasonal cycles (McAlary and McFarland, 1994). A record of the water temperature in the aquarium tank/delivery system during the deployment was not measured; however, aquarium tank temperatures measured from November 2014 to March 2015 show aquarium temperature ranges of ∼16–22°C (data not shown).

### Sample Collection, Scanning Electron Microscopy, and Geochemical Analyses

The HFMC was disassembled under sterile conditions for sample collection and discontinued after 60 days. FLOCS systems were disassembled after a 176-day deployment under sterile conditions. In addition, iron oxide precipitates at the bottom of the aquarium near the colonization experiments were also collected for cross-comparison to microbial communities in colonization samples (referred to as “microbial mat,” **Figure 2B**; **Table 1**). All collected samples were frozen immediately (-80°C) for subsequent nucleic acid extraction and microscopy analysis. At the end of the experiment, a total of 4-L of aquarium seawater was filtered onto a 0.2 μm mesh polycarbonate filter membrane and stored frozen (-80°C) for interrogation of the background microbial community from cove water (note that this community may have been influenced by aquarium conditions, although the relatively high flushing rate of the aquarium would minimize this). A decade-long study suggests that a stable euphotic zone core microbial community persists at the nearby San Pedro Ocean Time Series (Chow et al., 2013).

To examine the deposition of secondary minerals on the colonization materials, scanning electron microscopy (SEM) was performed using a Hitachi Model TM-1000 (WIES) at magnifications of 800–7000× immediately after collection of HFMC substrates and using a JSM-7001F analytical field emission scanning electron microscope at the Center for Electron Microscopy and Microanalysis at the University of Southern California. An ethanol dehydration series (70% for 12 h, 95% for 12 h, and 100% for 1 h), followed by oven drying (65°C) for 2 h, with no cell fixation procedure was followed prior to exposing samples to a vacuum.

Fluids (∼1.2 ml) contained within 1-m long sections of the chemical collection coil from the AF-FLOCS (**Figure 2C**) were expelled into acid-cleaned 1.5 ml plastic tubes following procedures outlined elsewhere (Wheat et al., 2011). Time stamps for individual samples are based on the number of samples, length of deployment and pumping rate. Temperature, salinity gradient, and surface area of the semipermeable membrane ultimately determine the pumping rate (Jannasch et al., 2004; Wheat et al., 2011). These pumps were exposed to air temperature fluctuations in the laboratory for the duration of the deployment, ranging across several degrees, which caused minor variations in the pumping rate over time. We recorded an average air temperature of 20.5°C in the laboratory throughout the course of the experiment, which would have resulted in an average pumping rate of ∼1 mL d^-1^. Samples were then stored and shipped at 4°C for Inductively Coupled Plasma-Optical Emission Spectrometry (ICP-OES) for major ions (Ca, Mg, K, S) and minor ions (Fe) following established protocols (Wheat et al., 2010).

### DNA Extraction and 16S rRNA Gene V4 Hypervariable Region Sequencing

Approximately 3 g of each substrate underwent total DNA extraction using the FastDNA^®^ Spin Kit for Soil (MP Biomedicals) following the manufacturer’s protocol (∼500 mg per extraction, six extraction tubes per sample). Each extraction was eluted in 75 μl of DES. Combined extracts were quantified in a NanoDrop 1000 Spectrophotometer and sent for library preparation and DNA sequencing by a commercial vendor (Molecular Research LP; MR DNA; Shallowater, TX, USA). Illumina MiSeq paired-end (2 × 250 bp) sequencing was performed targeting the V4 region of the 16S rRNA gene using the Earth Microbiome Project universal primers 515f (5′-GTG CCA GCM GCC GCG GTA A) and 806r (5′-GGA CTA CHV GGG TWT CTA AT) with 8-base barcodes on the forward primer (Caporaso et al., 2012). Briefly, amplification was carried out in a 30 cycle PCR using the HotStarTaq Plus Master Mix Kit (Qiagen, USA) under the following conditions as recommended elsewhere (Santiago-Rodriguez et al., 2015): 94°C for 3 min, followed by 28 cycles of 94°C for 30 s, 53°C for 40 s and 72°C for 1 min, after which a final elongation step at 72°C for 5 min was performed. After amplification, PCR products were analyzed on a 2% agarose gel via electrophoresis to determine the success of amplification and the relative intensity of bands. Multiple samples were pooled together in equal proportions and purified using calibrated Ampure XP beads. The pooled and purified PCR product was used to prepare the DNA library by following the Illumina TruSeq DNA library preparation protocol^2^.

### Sequencing Data Processing and Statistical Analysis

Sequence data curation and processing were performed with *mothur* v.1.34.4 (Schloss et al., 2009) following the *mothur* Illumina MiSeq Standard Operating Procedure (Kozich et al., 2013). In brief, paired reads were merged and any sequences with ambiguous base calls or homopolymers longer than 8 bp were culled. Merged reads were aligned to the *mothur*-recreated Silva SEED database from v119 (Yarza et al., 2010). Sequences were pre-clustered at a near 1% dissimilarity using the *pre.cluster* command with differences = 2. This process ranks sequences by abundance, then merges the most rare with the most abundant if they differ by only 2 bp as this has been shown to mitigate the generation of spurious sequences (Kozich et al., 2013). This pre-clustered dataset was, screened for chimeras, using the *de novo* mode of UCHIME (Edgar et al., 2011), which were then removed from any further processing and analysis. A distance matrix was generated for the remaining sequences and they were subsequently clustered into operational taxonomical units (OTUs) at 3% or less sequence dissimilarity using the average neighbor method. OTUs were taxonomically classified within *mothur* using the Ribosomal Database Project *release 9* dataset (Cole et al., 2014).

High abundance OTU sequences were aligned against the NCBI Bacteria/Archaea database using the blastn algorithm. High similarity near-full length sequences retrieved from this database were aligned with selected high abundance OTUs using MUSCLE (Edgar, 2004). A phylogenetic tree was generated using the UPGMA method, using 1000 bootstraps for branching support, and branches cladogram transformed with the *Geneious* software package (Kearse et al., 2012). Pairwise community comparisons for shared community composition and species richness were performed on subsampled datasets standardized to equal sizes (*n* = 11,763 for each sample) using the following *mothur* calculators: J_est_, Kulczynski, and Anderberg, for similarity in community membership analysis, in addition to the Bray–Curtis calculator, which analyzes community structure (Schloss, 2009). Results of each metric were compared to examine microbial community trends. As our data here were more driven by differences in abundances than by presence/absence, we utilized the Bray–Curtis dissimilarity in applicable downstream functions. Visualizations of the OTU abundance matrix were generated with *RStudio* version 0.98.1091 (Racine, 2012) using the packages *vegan* version 2.3-0 (Oksanen et al., 2015) and *rgl* version 0.95.1201. Principle components analysis was performed with the *prcomp()* function on a Bray–Curtis dissimilarity matrix calculated with the *vegsdist()* command. Sequence data were submitted to Genbank under Bioproject number PRJNA342057 and FASTA formatted sequences are available under the following accession numbers: KAHL01000001–KAHL01020009.

### Spectromicroscopy

Scanning transmission X-ray microscopy (STXM) analysis was conducted at the 5.3.2.2 beamline, Advanced Light Source, Berkeley, CA, USA (Kilcoyne et al., 2003). This beamline is well-suited for investigations of nano-sized natural particles composed of organic and inorganic carbon and iron oxide and oxyhydroxide minerals. STXM was used to collect three types of data: (1) transmission images of 10 μm^2^–1 mm^2^ areas, (2) elemental maps of C and O, and (3) C 1s and O 1s X-ray absorption near edge structure (XANES) spectra for points, lines, or areas (Toner et al., 2016). Elemental maps of C and O reveal morphology and the co-location of elements. Carbon 1s XANES spectra distinguish among organic and inorganic compounds while O 1s spectra are sensitive to several forms of Fe oxides and oxyhydroxides (Brandes et al., 2010; Chan et al., 2011; Bennett et al., 2014).

Incubated mineral chips from the NE-FLOCS-Pyrite and NE-FLOCS-Mild Steel samples (**Table 1**) were placed in clean tubes, gently rinsed with 18 MΩ grade deionized water (Milli-Q), and vortexed to suspend loosely adhered materials from the chips. Around 100 μL of the suspension was transferred to a new tube to dilute and wash any sea salt from the particles. Approximately 1 μL of suspension was deposited on a silicon nitride membrane and dried under ambient conditions. Once dry, samples were placed in the helium flushed STXM chamber. All data analysis—alignment of image stacks, principle component and cluster analysis, and normalization of spectra—was conducted using the freely available software *axis2000*^3^. Normalized spectra were compared to a reference database of C 1s and O 1s standards for species identification. Our C 1s reference database contains spectra of a lipid, PE lipid, BSA, agarose, and calcium carbonate. For O 1s we had access to reference spectra for various iron oxyhydroxides, but no iron oxide. A description of the reference materials for the C 1s and O 1s are published elsewhere (Toner et al., 2009, 2016; Chan et al., 2011). Distinct peak(s) and shape of O 1s iron oxides spectra were compared to data published elsewhere (Park et al., 2008).

## Results

### Visual Observations of the Experimental Progression

**FIGURE 3:**
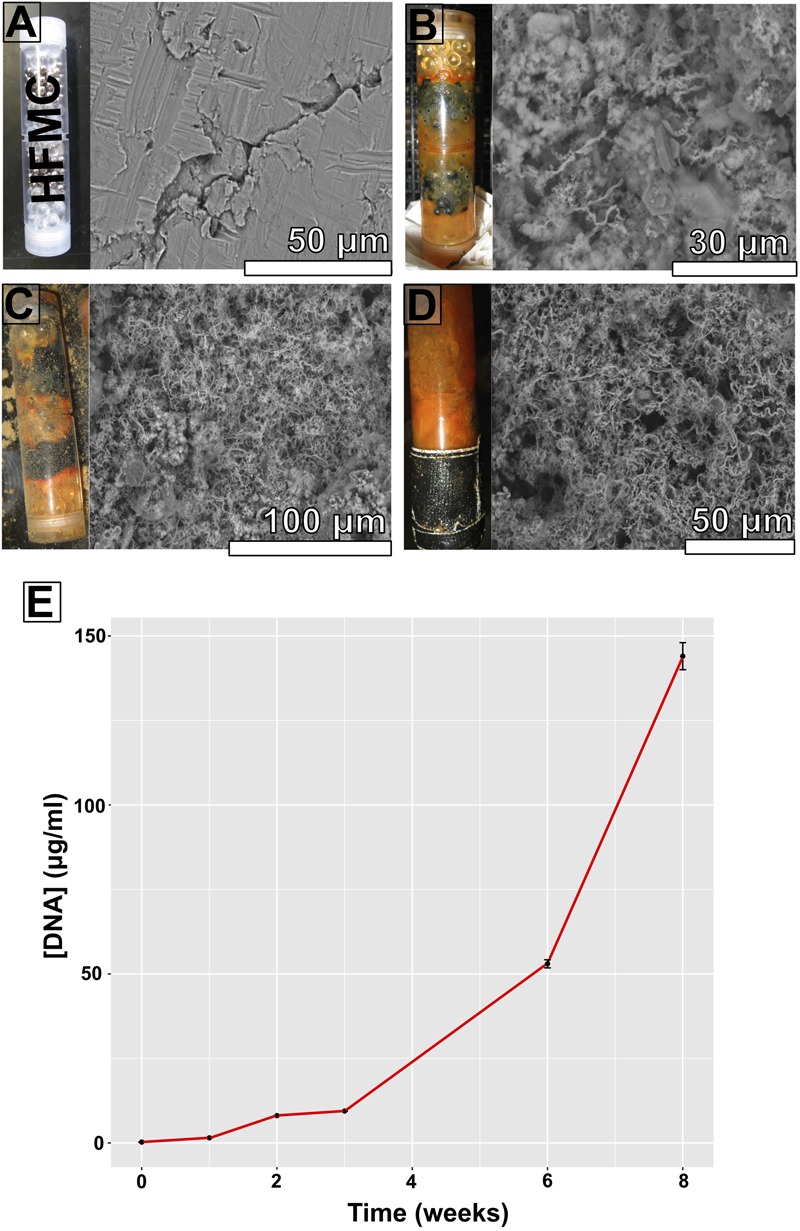
**High-flux microbial colonization colonization chamber photographs, corresponding scanning electron microscopy (SEM) images of substrate bioalteration (with corresponding scale bars), and DNA concentrations at specific time intervals spanning an 8-week period. (A)** Start of experiment; **(B)** 3 weeks of incubation; **(C)** 6 weeks of incubation; **(D)** 8 weeks of incubation; **(E)** DNA concentrations (in micrograms DNA per milliliter DNA extract) from ∼500 mg samples.

**FIGURE 4:**
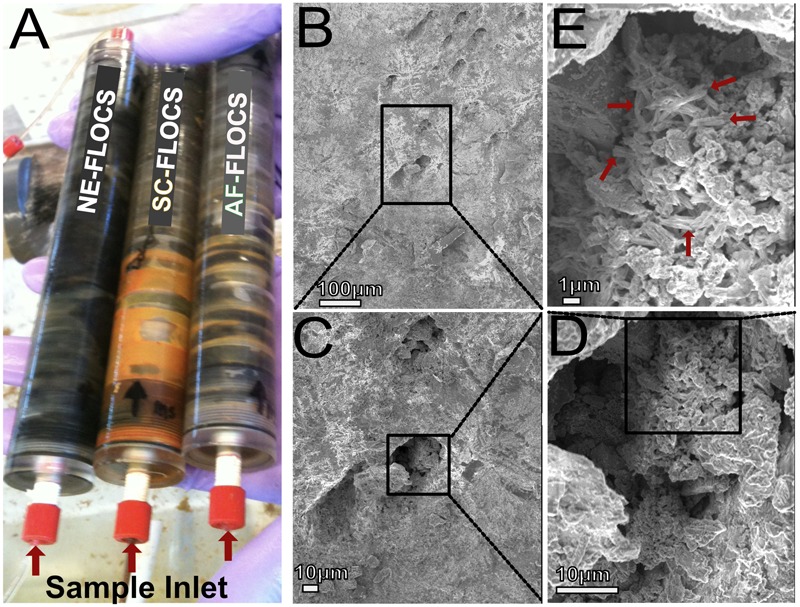
**(A)** Photograph of FLOCS chambers after 6 months of incubation; **(B–E)** SEM images of AF-FLOCS pyrite substrate surface pit at the end of the experiment, highlighting the presence of particles resembling structures known to be made by neutrophilic iron oxidizing bacteria.

The high flow (HFMC) experiment was subsampled and photographed periodically, revealing the steady build up of rust-colored particles in the colonization chamber over time (**Figures 3A–D**). The FLOCS colonization chambers exhibited visual changes related to metal corrosion (**Figure 4A**). The nutrient-enriched (NE-FLOCS) deployment was completely filled with black colored material and smelled strongly of hydrogen sulfide when opened, suggesting anoxic conditions inside the chamber at the time of collection. The active flow (AF-FLOCS) colonization chamber exhibited bands of rusty red and black zones that were more pronounced near the inlet on the mild steel substrate. The extent of rust accumulation was significantly lower in the active flow deployment, where active osmotic pumping of seawater was continuous, relative to stagnant condition (SC-FLOCS), where no osmotic pumping occurred. The stagnant condition exhibited profuse accumulations of rust-like material that was particularly accentuated over the mild steel cassettes located near the inlet of the colonization chamber. This rust-like precipitate exited the system via the inlet and accumulated on the aquarium bottom (see **Figure 2B** for schematic), creating an iron microbial mat-like deposit with iron oxides at the seawater interface and an apparent black anoxic zone a few millimeters below.

### SEM Imaging of Colonized Substrates

**FIGURE 5:**
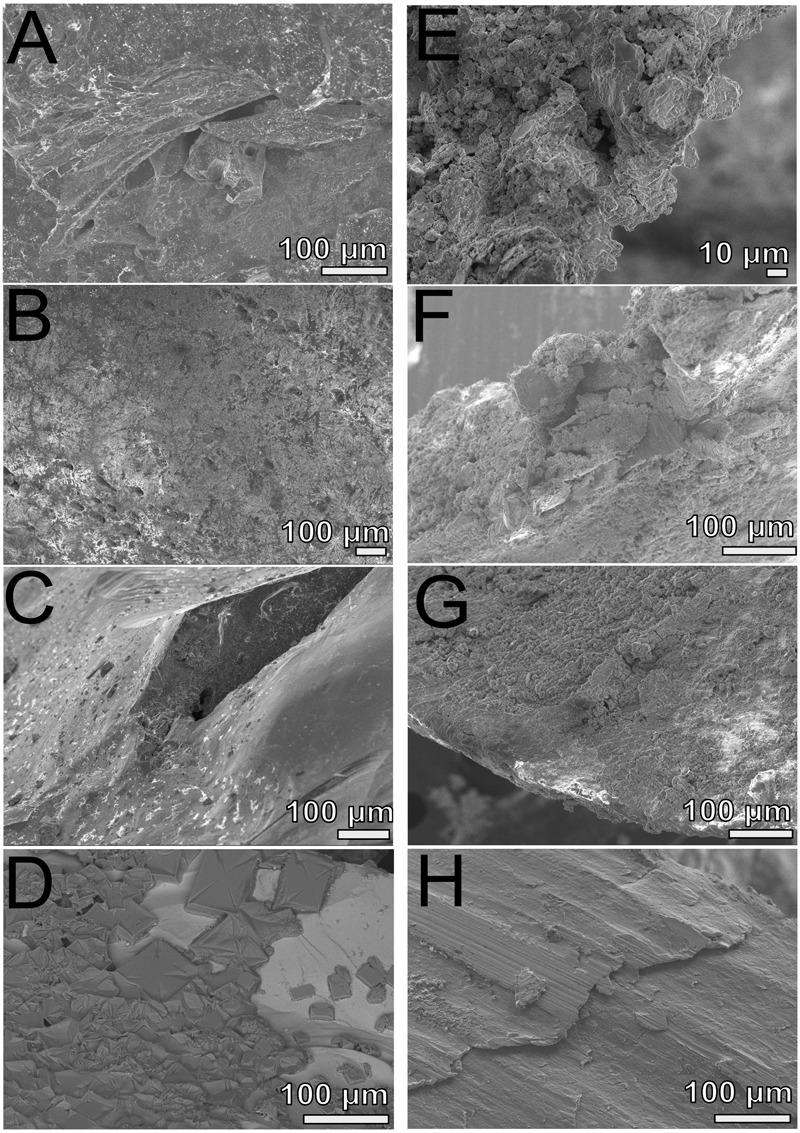
**Scanning electron microscopy analyses of FLOCS substrates (pyrite, left panels; mild steel, right panels) after 6-months of incubation. (A)** NE-FLOCS pyrite; **(B)** AF-FLOCS pyrite; **(C)** SC-FLOCS pyrite; **(D)** sterile pyrite surface, not incubated; **(E)** NE-FLOCS mild steel; **(F)** AF-FLOCS mild steel; **(G)** SC-FLOCS mild steel; **(H)** sterile mild steel, not incubated.

Scanning electron microscopy imaging of colonized mild steel substrates from the high flow experiment at 2 or 3 week intervals revealed an increasing abundance of twisted stalk particles (**Figures 3A–D**) that followed a steady increase in the DNA concentration recovered from nucleic acid extractions from ∼500 mg of sample (**Figure 3E**). Similar twisted stalk particles are known to be biogenic precipitates of neutrophilic FeOB (Chan et al., 2011). In contrast, the mild steel and pyrite substrates from the FLOCS experiments did not reveal such abundances of twisted stalk particles, but they did show evidence of amorphous alteration (**Figure 5**). Overall, the mild steel substrates from all three FLOCS treatments (**Figures 5D,F,G**) had more evidence of alteration than the pyrite substrates (**Figures 5A–C**). The stagnant condition pyrite substrate (**Figure 5C**) exhibited far less alteration as compared to the pyrite from the active-flow FLOCS (**Figure 5B**), whereas the mild steel substrate from the stagnant condition (**Figure 5G**) had similar levels of alteration to the mild steel substrates from the other treatments (**Figures 5E,F**). In some cases, surface pits in the colonized minerals from the FLOCS appeared to contain structures resembling degraded twisted stalks (**Figures 4B–E**).

#### Bacterial Diversity and Phylogeny

DNA was extracted from all deployments (**Table 1**). Using a ∼500 mg sample (wet weight including iron filings), extracted DNA yields ranged in concentration from ∼10–100 ng μl^-1^ for FLOCS (data not shown), with a clear increase in DNA concentration in the high flow experiment over time (**Figure 3E**). The V4 region of the 16S rRNA gene was amplified and sequenced from the environmental DNA extracts from all samples except for the high flow incubation, as these samples were lost during a laboratory transition.

**FIGURE 6:**
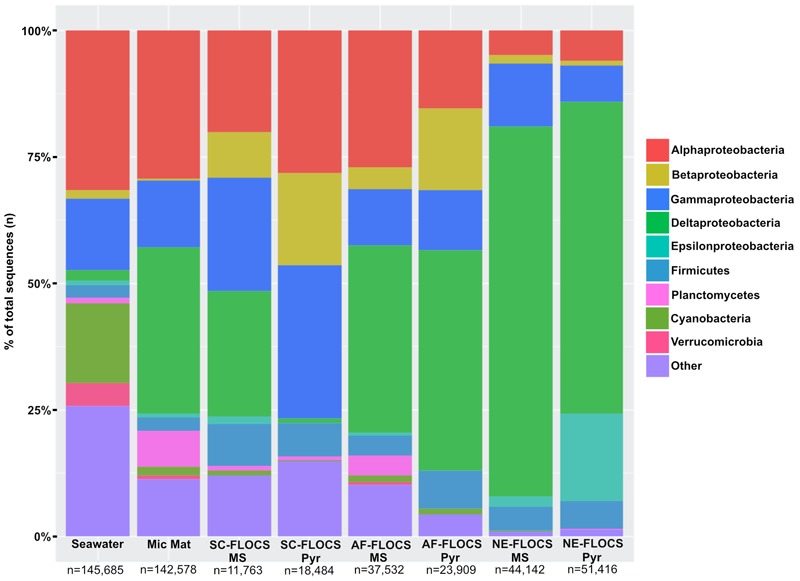
**Taxonomic percent breakdown at Phylum-level resolution (Class-level for the Proteobacteria) of processed Illumina V4 16S rRNA gene sequences.** Total number of reads for each sample is depicted under sample name. Pyr, pyrite; MS, mild steel. Zetaproteobacteria sequences were not observed in the data, and no sequencing was conducted on the HFMC samples.

**FIGURE 7:**
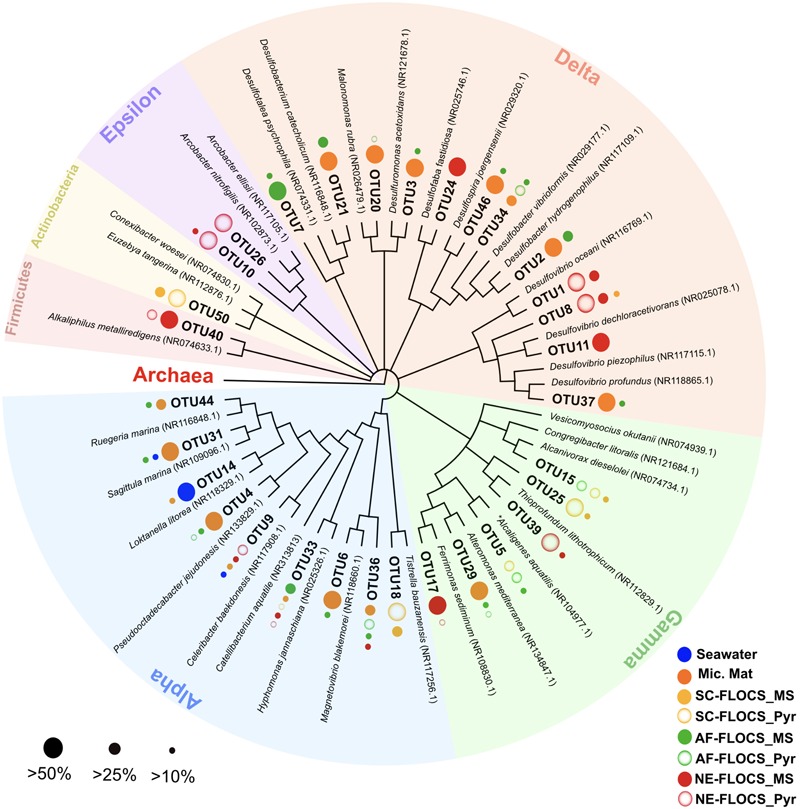
**Phylogenetic overview of 31 high abundance bacterial OTUs representing 50% of all sequences recovered in this study.** Dots next to OTU branches represent the OTU sequence percent prevalence (dot size) in samples for which that particular OTU comprised more than 10% of all sequence reads (color code). ^∗^Betaproteobacterium branching within the Gammaproteobacteria.

Processed V4 region 16S rRNA gene data resulted in 475,509 total bacterial sequences, with a range of 11,763–145,685 sequences per sample (**Figure 6**). All sequences were subsampled to the lowest sample depth of 11,763 prior to comparative analyses. These sequences grouped into 208–14,533 OTUs per sample (seawater filtrate: 6,117; microbial mat: 14,533; SC-FLOCS mild steel: 267; SC-FLOCS pyrite: 356; AF-FLOCS mild steel: 784; AF-FLOCS pyrite: 208; NE-FLOCS mild steel: 366; and NE-FLOCS pyrite: 377; data not shown). The aquarium seawater exhibited the highest taxonomic diversity and was dominated by the Proteobacteria phylum, particularly the Alpha- and Gamma-proteobacteria classes, which accounted for 45.3% of all sequences (**Figures 6** and **7**). The microbial mat and SC-FLOCS mild steel substrate contained microbial communities primarily comprised of Alpha-, Beta-, Gamma- and Delta-Proteobacteria, with the major difference being a disproportional presence of Beta- and Delta-Proteobacteria sequences, where the later are more dominant in the microbial mat sample (30.1% vs. 24.7%) while the former are more heavily represented in the SC-FLOCS mild steel substrate (8.9% vs. 0.4%, **Table 2**). The SC-FLOCS pyrite is similar in taxon distribution to the SC-FLOCS mild steel substrate but had substantially less Deltaproteobacteria sequences (0.9% in pyrite vs. 24.7% in mild steel, **Figure 6**). The AF-FLOCS pyrite and mild steel sequence taxonomy distributions are also similar to each other with slight differences in the relative distributions of Betaproteobacteria (**Table 2**). The NE-FLOCS mild steel and pyrite substrates are taxonomically similar with the exception being the Epsilonproteobacteria class distribution, which comprised 17.1% of pyrite sequences and only 2.0% of sequences from mild steel (**Figure 6**). A high number of sequences recovered from AF- and NE-FLOCS classified as Deltaproteobacteria. NE-FLOCS substrates had the highest number of Epsilonproteobacteria recovered (**Figure 6**; **Table 2**).

**Table 2:** Percent abundance of the Beta-, Delta-, and Epsilon-proteobacteria sequences, as well as select genera or orders within those Proteobacteria classes, for the colonized FLOCS materials, microbial mat from the bottom of the aquarium, and surrounding background seawater.

	NE-FLOCS-Pyr	NE-FLOCS-MS	AF-FLOCS-Pyr	AF-FLOCS-MS	SC-FLOCS-Pyr	SC-FLOCS-MS	MicMat	Seawater
**Betaproteobacteria (% of all sequences)**	1.0	1.6	16	4.2	18	7.0	0.4	1.6
*Burkholderiales* (% of Betaproteobacteria)	98	98	100	91	100	100	35	4
**Deltaproteobacteria (% of all sequences)**	62	73	44	37	0.8	25	33	2
*Desulfovibrio* (% of all Deltaproteobacteria)	98	88	35	7	26	96	5	3
*Desulfobacter* (% of all Deltaproteobacteria)	<0.1	<0.1	8	37	<0.1	0.3	36	2
**Epsilonproteobacteria (% of all sequences)**	17	2	<0.1	0.1	<0.1	1.4	0.8	1.0
*Arcobacter* (% of all Epsilonproteobacteria)	100	100	83	96	77	1	46	59

*Sequencing was not performed on the HFMC sample. Alpha- and Gamma-proteobacteria sequences are not summarized here (see **Figure 2**), and Zetaproteobacteria sequences were not detected.*

A phylogenetic overview emphasizing 31 high abundance OTUs (representing 50% of all sequences) recovered from FLOCS substrates shows a wide range of Proteobacterial diversity (**Figure 7**). The three most dominant OTUs fell within the Deltaproteobacteria class, an anaerobic cohort capable of sulfate (*Desulfovibrio, Desulfobacter*) and sulfur (*Desulfuromonas*)-reduction. OTU1, the most prevalent OTU in this study, is closely related (99% sequence similarity) to *Desulfovibrio oceani*, with high abundance in both NE-FLOCS substrates and low abundance in all other samples (**Figure 7**; **Table 2**). The next two most dominant OTUs, OTU2 and OTU3, are closely related (99 and 97% sequence similarity, respectively) to *Desulfovibrio hydrogenophilus* and *Desulfuromonas acetoxidans*, respectively (**Figure 7**). OTU 17, recovered primarily from NE-FLOCS substrates (**Figure 7**), groups closely (100% sequence similarity) with *Ferrimonas sediminum*, a Gammaproteobacterium capable of Fe(III) oxyhydroxide reduction (Ji et al., 2013). OTU 39 is a Gammaproteobacterium related (100% sequence similarity) to *Thioprofundum lithotrophicum*, a deep-sea hydrothermal sulfur-oxidizing obligate chemolithoautotroph (Mori et al., 2011), observed in disproportionally high numbers in the NE-FLOCS substrates exclusively (**Figure 7**). Additionally, OTUs 26 and 10, both Epsilonproteobacteria, grouped within the sulfur-oxidizing *Arcobacter* genus (Pati et al., 2010; Sasi Jyothsna et al., 2013) and were predominantly found on NE-FLOCS substrates. OTU4, present in high numbers in both AF-FLOCS substrates and a microbial mat while being extremely low in all other incubation conditions and seawater, was closely related (98% sequence similarity) to *Octadecabacter jejudonensis*, a novel nitrate-reducing marine Alphaproteobacterial isolate from Jeju Island, South Korea (Park and Yoon, 2014). OTU 5, present in high abundance in all FLOCS-substrates relative to a microbial mat and seawater, is related (99% sequence similarity) to a nitrate-dissimilating Betaproteobacterium (*Alcaligenes aquatilis*) previously isolated from Teutonic estuary sediments and a North American salt marsh (Van Trappen et al., 2005).

#### Community Membership Statistical Analyses

**FIGURE 8:**
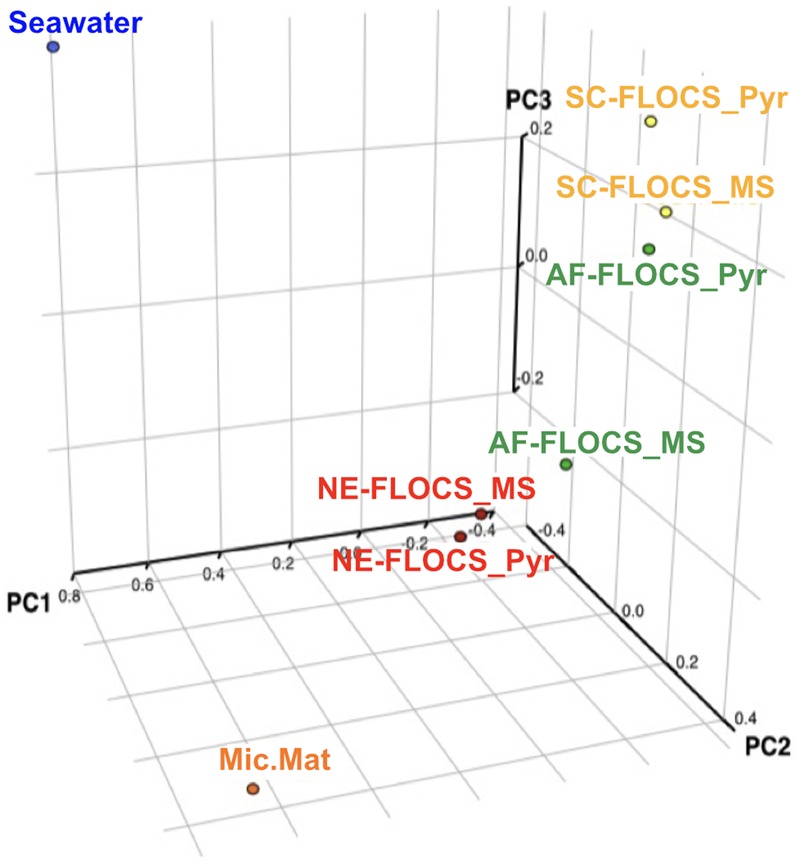
**Principal component analyses (PCA) of 16S rRNA gene sequences recovered from each FLOCS substrate, background seawater, and a microbial mat recovered from the aquarium tank bottom**.

The Bray–Curtis dissimilarity index, used to quantify hierarchical clustering of community membership similarity, consistently clustered both Enriched-FLOCS pyrite and mild steel substrates as most similar to each other relative to other samples (data not shown). Principal component analysis (PCA) shows distinct separation of the seawater and microbial mat samples along two axes with close placement of the Enriched-FLOCS mild steel and pyrite substrate and FLOCS enrichment type separated along a third axis (**Figure 8**). Overall, samples are grouped as a function of FLOCS deployment condition rather than substrate (pyrite vs. mild steel) type (**Figure 8**).

### Geochemical Analysis

**FIGURE 9:**
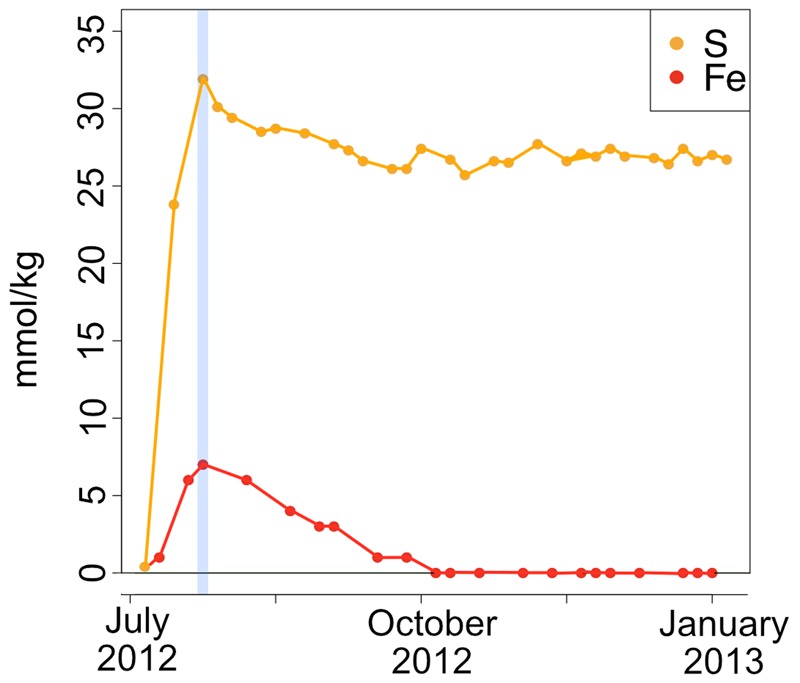
**Fe and S concentrations (in millimole per kg fluid) in the AF-FLOCS experiment plotted as a function of time from initial conditions (July 2012) to experiment end (January 2013).** The blue vertical line indicates the Fe and S co-maxima.

A time series of the concentrations of major and minor ions in seawater was analyzed from the eﬄuent from the AF-FLOCS only, as other experiments did not have the acid preservation of the fluid samples for analysis, as recommended (Wheat et al., 2011). All conservative, non-reactive major ions (i.e., Ca, Mg, K) exhibited steady concentrations throughout the experiment (data not shown). Dissolved sulfur, which could represent the sulfate ion from seawater or hydrogen sulfide oxidized to sulfate, exhibited an initial spike in concentration to 31.9 mmol kg^-1^ as compared to background seawater concentrations of 28 mmol kg^-1^, likely reflecting leaching of sulfide from the pyrite in the colonization chamber (**Figure 9**). For the remainder of the experiment, S remains fairly steady. Total iron also spiked at the beginning of the experiment, followed by a gradual decline to concentrations below the limit of detection after 3 months of incubation (**Figure 9**).

### Spectromicroscopy Analyses

**FIGURE 10:**
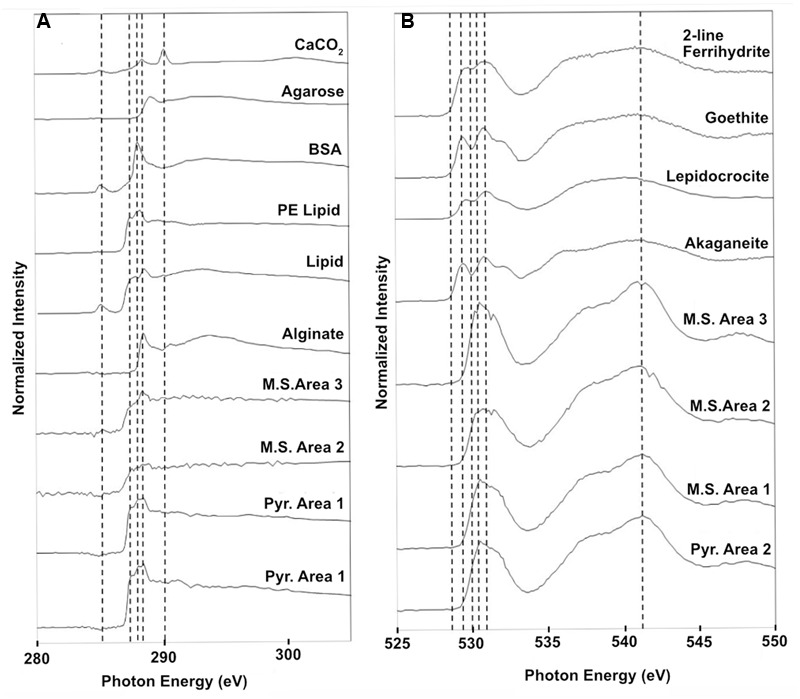
**Normalized STXM three-dimensional stacks of particles on the NE-FLOCS substrates; **(A)** Carbon 1s XANES spectra found on both pyrite and mild steel along with reference spectra; **(B)** Oxygen 1s XANES spectra for both pyrite and mild steel along with reference spectra for 2-line ferrihydrite, goethite, lepidocrocite, and akaganeite.** Vertical dashed lines are for reference in aligning spectral features across samples.

**FIGURE 11:**
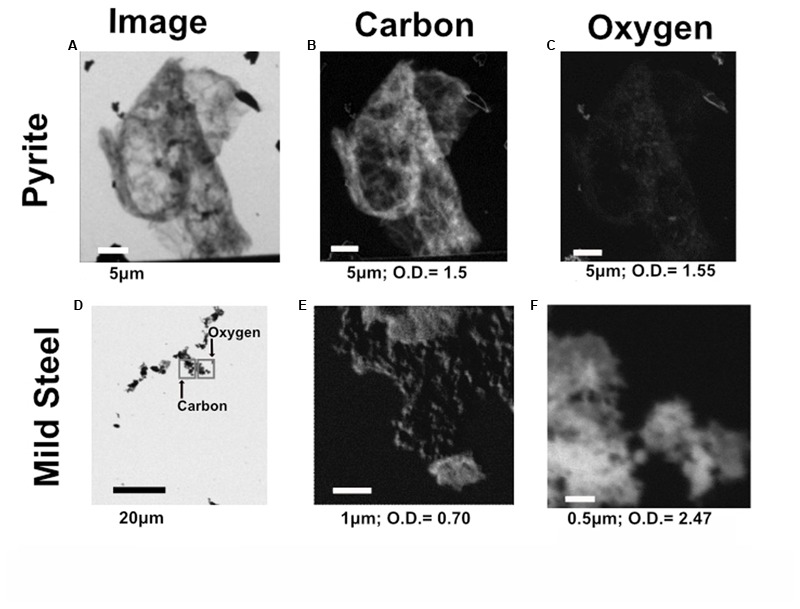
**Representative C and O elemental maps from both pyrite and mild steel within the NE-FLOCS. (A)** Transmission image of representative particle on pyrite substrate; **(B)** C distribution map of particle on pyrite substrate; **(C)** O distribution map of particle on pyrite substrate. **(D)** Transmission image of representative particle on mild steel substrate, with gray squares representing where the elemental maps were collected for both C and O to avoid beam damage; **(E)** C distribution map of particle on mild steel substrate: **(F)** O distribution map of particle on mild steel substrate. Length of scale bar in each image indicated below image, along with maximum optical density values for each elemental map.

Scanning transmission X-ray microscopy analysis was performed on the NE-FLOCS only, due to constraints in beamline availability. The C1s XANES spectra are consistent with organic C, and the best spectral match with our reference database is a lipid compound [phosphatidylethanolamine (PE)]. It is unlikely that this organic matter was derived from the marine broth residue, as the samples were serially rinsed during preparation. In some instances, C was observed but the spectra lack identifying features (**Figure 10A**); this could be caused by a low concentration of C in an optically thick sample. Oxygen 1s XANES spectra for both the mild steel and pyrite did not match any spectra in our reference database. Instead, the spectral shapes and peaks best aligned with magnetite (Fe_3_O_4_) nanoparticles reported in Park et al. (2008) (**Figure 10B**). Two distinct peaks, ∼531 and ∼541 eV, were observed in magnetite nanoparticles that were distinct in shape and resolution from bulk magnetite. The first peak at ∼531 eV is broad in shape with only one absorption peak, with the second peak having a low energy shoulder with a main peak at ∼541 eV. These features are consistent with the electron configuration of Fe interacting with the oxygen in magnetite (Park et al., 2008). For both the pyrite and mild steel substrates, elemental maps and three-dimensional stacks were analyzed on at least three representative particles. C 1s and O 1s were measured to investigate speciation and understand particle morphology. Sample elemental maps indicated homogenous carbon-rich matrices surrounding pyrite particles and floccose distinct particles, rich in both carbon and oxygen, present on mild steel (**Figure 11**). For both the pyrite and mild steel incubations, ubiquitous carbon-rich matrices were observed.

## Discussion

In an effort to assess the ecological response of the endemic pelagic microbial community present in southern California coastal waters to Fe-enrichment under eutrophic conditions, we deployed microbial colonization chambers in aquarium tanks in Santa Catalina Island supplied with a constant influx of nearby cove water. The extent of biocorrosion of ferruginous substrates was analyzed under the physical, nutritional, and temporal variables provided by deployment configurations. Our data corroborate the potential initial involvement of FeOB, and subsequent microbial succession to cohorts of S-cycling Epsilon and Delta-Proteobacteria, in conditions mimicking the severe form of MIC known as ALWC, as postulated by others (Beech and Campbell, 2008; Dang et al., 2011). A 2-month incubation of mild steel under high flow, oxic conditions led to surface weathering and biomineralization resembling that of marine FeOB. Our microscopy results corroborate findings of a recent study of mild steel colonization by Zetaproteobacteria in West Boothbay Harbor, Maine (McBeth and Emerson, 2016). Six months of nutrient stimulation in colonization chambers results in biologically altered surfaces on both mild steel and pyrite, with differences in bacterial community composition when compared to non-nutritionally enhanced substrates, but scant evidence of Zetaproteobacteria-like biominerals (i.e., twisted stalks). Interestingly, no sequences classified as Zetaproteobacteria were recovered from any 6-month deployment samples. We speculate, based on microscopy evidence for 2-month deployments and molecular, geochemical and spectromicroscopy evidence for 6-month deployments, on the possible ecological succession mechanisms that may result in ALWC near a port of global economic importance.

### Geochemical Implications for Biological Fe Oxidation

During high Fe conditions in the FLOCS experiments (first month of the deployment, **Figure 9**), Fe and oxygen gradients at the interphase of both mild steel and, possibly, pyrite may have recruited FeOB as observed in our 2-month study (discussed below). Therefore, during this initial incubation stage, sulfate rises above its normal concentration in seawater (background sulfate + substrate S-species leachate, **Figure 9**), since microorganisms present in the colonization chamber, as suggested by microscopy results (**Figures 3A–D**), likely belong to Fe-rather than S-utilizing guilds. The decrease of Fe begins after approximately 4 weeks (**Figure 9**). This observation can be explained by extensive biological Fe-oxidation activity preventing further release of Fe^2+^ from substrates, ultimately disturbing the Fe and oxygen gradients necessary to favor the metabolism of FeOB (Edwards et al., 2003b; Emerson et al., 2010). We hypothesize that, in long-term FLOCS incubations, early FeOB proliferation leads to a relatively oxygen depleted niche (due to FeOB metabolism and slow FLOCS osmotic pump rates) that, as observed by co-stabilization of S within the timeframe of Fe depletion (**Figure 9**), begins to favor anaerobic S-cycling microbial cohorts, whose presence and predominance we confirmed on FLOCS substrates at the end of a 6-month deployment (**Figure 6**). Additionally, H_2_S, the product of S-reduction potentially occurring in anoxic microniches early in FLOCS, can react with Fe^2+^ and form ferrous sulfide, effectively removing both S and Fe species from solution and explaining their co-maxima followed by stabilization to background seawater levels (**Figure 9**).

### Colonization by Iron Oxidizing Bacteria

#### Two-month Incubations: Short-Term Mild Steel Biocorrosion

Electron microscopy analyses shows that, over an 8-week period (**Figure 3**), substrate surface alterations increased in magnitude and strongly resembled biomineralized structures produced by the Zeta-proteobacterium *Mariprofundus ferrooxidans* (Emerson et al., 2010; Chan et al., 2011; McBeth et al., 2011). Our observations are consistent with previously reported timeframes for FeOB biomineral deposit electron microscopy-based detection ranging from 2 weeks for incubations of mild steel coupons off Boothbay Harbor, Maine (McBeth et al., 2011) to 2 months for naturally occurring mineral sulfides at the Endeavor segment of the Juan de Fuca Ridge (Edwards et al., 2003b), implying that marine FeOB, as observed in other locations, colonize ferruginous substrates in waters near the port of Los Angeles. Molecular samples for this incubation were lost during field transport.

#### Six-month FLOCS Incubations: Long-Term Mild Steel and Pyrite Biocorrosion

Electron microscopy analysis of FLOCS sleeve substrates revealed different degrees of surface alteration corresponding to colonization substrate (mild steel > pyrite) and FLOCS deployment (NE-FLOCS > AF-FLOCS > SC-FLOCS; **Figure 5**). FLOCS substrates deployed for 6 months do not show twisted stalks on surfaces as observed in 2–8 week incubations (**Figures 3B–E**). Close examination of surface pits, particularly on the pyrite substrates from the AF-FLOCS, revealed encrustations of structures, ∼1 μm in width (**Figures 4B–E**), that ostensibly structurally resemble degraded twisted stalks observed during our 2 month incubation study (**Figures 3A–D**). Interestingly, a parallel study to our own identified Zetaproteobacteria in an iron sulfide mineral incubated *in situ* at our study site (Barco, RA, unpublished data). A pit-specific colonization strategy may grant neutrophilic FeOB a biofilm encrusted microaerophilic micro-niche leading to partial protection from initial advective and subsequently diffusive exchange with excess oxygen (Edwards et al., 2003b). We speculate that morphologically distinct particles (∼1 μm diameter) exclusively localized within various surface pits may be degraded fossilized remnants of a once active FeOB community on the pyrite surfaces incubated in AF-FLOCS. FeOB proliferation, in addition to slow osmotic pump rates, could be responsible for the relative depletion of oxygen within FLOCS chambers, resulting in the recruitment of anaerobic cohorts that may electrochemically corrode biomineralized Fe(III) of twisted FeOOH stalks (Emerson, 2009; Langley et al., 2009), as further discussed below. Such a process would explain the absence of structures with twisted stalk morphology on SEM surveys of 6-month FLOCS incubations (**Figure 5**), and begin recruitment of anaerobic S-cycling prokaryotes [as suggested by molecular evidence (**Figures 6** and **7**)], implying a biologically driven temporal coupling of S and Fe redox cycling for our geochemical data (**Figure 9**).

#### Neutrophilic FeOB Absent from FLOCS Molecular Survey

Neutrophilic FeOB have been particularly elusive when cultivation-independent methods have been employed for their detection in previous investigations involving *in situ* deep-sea mineral weathering (Thorseth et al., 2001; Edwards et al., 2003a; Orcutt et al., 2011; Baquiran et al., 2016). Our sequencing efforts at high taxonomic resolution on 6-month FLOCS colonization experiments failed to detect phylotypes associated with known neutrophilic FeOB, such as the Zetaproteobacteria. Instead, incubations were dominated by members of the Delta- and Epsilon-Proteobacterial classes for the AF-FLOCS and NE-FLOCS deployments, respectively, while SC-FLOCS substrates hosted Gamma-Proteobacterial sequences in high proportions (**Figure 6**). Interestingly, we observed the genera *Marinobacter* and *Pseudoalteromonas*, other potential marine FeOB, only in extremely low relative abundance (<0.01%) in both AF-FLOCS substrates and NE-FLOCS pyrite, exclusively (data not shown). Regarding the potential for fresh water FeOBs in our coastal incubations, a single sequence recovered from NE-FLOCS pyrite was classified as belonging to the Gallionellaceae Betaproteobacteria family, whereas other known fresh water FeOB genera such as *Leptothrix, Ferritrophicum*, and *Siderocapsa* (Emerson et al., 2010) were either completely absent or beyond taxonomic resolution in our dataset. Interestingly, the Gallionellaceae family have also been linked to colonization of deep marine sulfides (Kato et al., 2009; Li et al., 2012; Sylvan et al., 2012). PCA show robust statistical groupings based on FLOCS deployment condition (**Figure 8**), indicating that nutritional supplementation in colonization chambers (e.g., NE- vs. AF-FLOCS) played a stronger role than substrate type (mild steel vs. pyrite) in influencing microbial community membership similarity. We stress that our molecular survey (**Figure 6**) is a synoptic inference into the microbial community composition and structure at the end of the 6-month experiments and cannot address the plausible earlier presence of an active FeOB-like microbial community on FLOCS substrates. It is possible that, due to the wide phylogenetic distribution of lithoautotrophic FeOB in the environment (Edwards et al., 2004; Emerson et al., 2010), there may be Fe-oxidizing lithoautotrophic phylotypes in our data closely aligned, based on 16S rRNA gene similarity, with cultured heterotrophic representatives.

#### Spectromicroscopy

Similarities with the reference spectra and our carbon-rich matrices in the NE-FLOCS include line shape and amplitude with PE lipids (**Figure 10A**). PE lipids play important structural and transport roles in bacterial membranes and have been found to destabilize protein–lipid contacts (Scarlata and Gruner, 1997). The presence of PE lipids, as integral components of extracellular polymeric substances (EPSs), on NE-FLOCS substrates can be interpreted as indicative of microbiological activity. Oxygen XANES spectra on both the mild steel and pyrite (**Figure 10B**) are consistent with peaks measured for magnetite nanoparticles (Park et al., 2008). The presence of magnetite, the product of microbial dissimilatory Fe(III) reduction, as detailed by others (Kappler and Straub, 2005; Weber et al., 2006), provides evidence for potential Fe(III) reduction activity in the NE-FLOCS system further discussed below.

### Potential for Fe and S Cycling on Ferruginous Substrates under Eutrophic Conditions

#### Fe Oxidation in NE-FLOCS

Anaerobic conditions present in the NE-FLOCS during collection prohibited redox gradients necessary for the metabolism of neutrophilic microaerophilic FeOB. We therefore expected iron reduction rather than oxidation at the terminal stages of the incubation and sample collection. However; it is worth noting the dominance of the *Burkholderiales* order (**Table 2**) in the Beta-Proteobacterial sequences recovered from all FLOCS substrates (>98%) relative to Beta-Proteobacterial sequences recovered from background seawater (3.9%) and a microbial mat collected from the aquarium tank (35%). Despite representing more than 10% of total sequences in only two FLOCS substrates (**Figure 6**), the dominance of a single taxonomic order within this Proteobacterial class is of interest for nitrite reduction and, potentially, Fe oxidation in our incubations. OTU5 a high abundance taxon recovered primarily from SC and AF-FLOCS substrates (**Figure 7**), grouped close to *Alcaligenes aquatilis* a nitrite-reducing member of the *Burkholderiales* (Van Trappen et al., 2005). Recently, a *Burkholderiales* strain (GJ-E10), isolated from an acidic river in Japan, has been shown to be an iron-oxidizing chemolithoautotrophic bacterium (Fukushima et al., 2015). These results encourage further investigation of the potential involvement of the *Burkholderiales* order in Fe^2+^ oxidation of ferruginous substrates under eutrophic conditions. To our knowledge, this is the first time that *Burkholderiales* (potentially acidophilic FeOB) have been detected in marine ferruginous substrate enrichment experiments.

#### Fe Reduction in NE-FLOCS

In regards to possible Fe reduction, OTU40, recovered primarily from NE-FLOCS, grouped closely with *Alkaliphilus metalliredigens* (**Figure 7**), an Fe(III)-citrate reducing species isolated from borax-contaminated leachate (Ye et al., 2004). OTU3, found in AF-FLOCS and the microbial mat sample, is a close relative of *Desulfuromonas acetoxidans* (**Figure 6**), a species known for coupling organic compound oxidation with dissimilatory Fe(III) reduction and/or the reduction of S^o^ (Roden and Lovley, 1993). OTU17, a high abundance sequence from this study recovered almost exclusively from NE-FLOCS substrates, is a close relative of *Ferrimonas sediminum* (**Figure 6**), a species capable of using Fe(III)-oxyhydroxide minerals as terminal e^-^ acceptors (Ji et al., 2013). We propose that “twisted stalks” are absent in 6-month deployments due to (i) an oxic to anoxic redox regime shift halting their biological production and (ii) their subsequent dissolution catalyzed by bacterial dissimilatory Fe(III)-oxyhydroxide reduction. Microbial Fe reduction is further corroborated by spectromicroscopic identification of magnetite on NE-FLOCS substrates, as previously discussed (see Spectromicroscopy).

#### Sulfate Reduction in NE-FLOCS

Sulfate reducing bacteria are a diverse obligate anaerobic guild of the Deltaproteobacteria class sharing only a common final electron acceptor for anaerobic respiration (Muyzer and Stams, 2008). A high number of Deltaproteobacteria sequences from NE- and AF-FLOCS (**Figure 5**) were taxonomically resolved to the genus-level and classified as *Desulfovibrio* spp. (**Table 2**; **Figure 6**). The highest numbers of *Desulfovibrio* sequences were recovered from the NE-FLOCS system (**Table 2**) that, as suggested by smell of hydrogen sulfide during recovery, we assume was anoxic. OTUs 1, 8, and 11 are high abundance sequences, recovered nearly exclusively from NE-FLOCS, closely related to the following *Desulfovibrio* species: *D. oceani* sp., *D. piezophilus* sp., and *D. dechloracetivorans*, respectively (**Figure 6**). *D. oceani* has been isolated from the oxygen minimum zone off the Peruvian coast and characterized as an SRB (Finster and Kjeldsen, 2010) and *D. piezophilus* is an SRB from Mediterranean deep (1693 m) waters (Khelaifia et al., 2011). Interestingly, *D. dechloracetivorans*, isolated from the San Francisco Bay, is a SRB with the unusual capacity for *ortho*-chlorophenol reductive dechlorination and may be a proxy for halogenated pollutants at our study site (Sun et al., 2000). The most common taxon recovered from AF-FLOCS (OTU7) is closely related to *Desulfotalea psychrophila*, a Deltaproteobacterium isolated from sediment off the Norwegian Arctic Island of Svalbard, whose genome bears genes for sulfate and thiosulfate reduction (Rabus et al., 2004) and has been previously suggested to perform dissimilatory Fe(III) reduction (Knoblauch et al., 1999). Lower abundances of *Desulfovibrio* spp. were recovered from the AF-FLOCS substrates suggesting, as supported by PCA (**Figure 8**), that nutritional enrichment plays a more determinant role than substrate type in the ecological recruitment of Deltaproteobacteria on ferruginous substrates. Overall, the *Desulfovibrio* genus, present in all FLOCS deployment substrates but disproportionately numerous under nutrient enrichment, has cultivated representatives capable of sulfate reduction coupled to anaerobic iron oxidation (Dihn et al., 2004) and is often implicated in SRB-mediated corrosion of steel in anoxic environments (Kakooei et al., 2012), as further discussed below.

#### Epsilonproteobacteria in NE-FLOCS: Sulfide Oxidation and Potential Denitrification

Nutritional Enrichment FLOCS yielded the highest proportion of sequences classified as Epsilonproteobacteria (**Figure 6**). Members of the Epsilonproteobacteria class are considered ubiquitous in sulfidic terrestrial and marine environments and play important roles as chemolithoautotrophic inhabitants of deep-sea hydrothermal vent systems where they are responsible for light-independent carbon fixation coupled with sulfide oxidation (Campbell et al., 2006). The vast majority of these sequences were taxonomically resolved to the genus *Arcobacter* (**Table 2**). *Arcobacter* spp. are known sulfide oxidizers and can fix carbon via the rTCA cycle (Hugler et al., 2005). This genus has been implicated in dissimilatory Fe and Mn-reduction and, at least one cultured isolate, is capable of extracellular electron transfer to an anodic electrode (Fedorovich et al., 2009). *Arcobacter* spp. may be indirectly involved in MIC by: (i) H_2_S (a product of SRB activity) detoxification and (ii) facilitating SRB and FRB activity by actively removing O_2_ (De Gusseme et al., 2009). *Arcobacter* spp. were recently reported in seafloor-deployed FLOCS experiments at sub-seafloor observatories in the Juan de Fuca ridge flank (Baquiran et al., 2016), highlighting their physiological versatility and remarkable ecological range. The majority of *Arcobacter* spp.-related sequences in this study were represented by OTUs 10 and 26 which are closely related to *Arcobacter ellisii*, a mussel isolate (Figueras et al., 2011), and *Arcobacter nitrofigilis*, a free-living and symbiotic partner of marine invertebrates capable of nitrogen fixation (Pati et al., 2010), respectively (**Figure 7**). Recently, genomic analysis of *Arcobacter anaerophilus* IR-1, a North Sea oilfield isolate, revealed an incomplete denitrification pathway where the reduction of nitrate to nitrite (a corrosive metabolite), rather than ammonium, directly implicates the *Arcobacter* genus in MIC (Roalkvam et al., 2015). We speculate that incomplete denitrification, possibly performed by *Arcobacter* spp. in our experiments, resulting in nitrite production, may concomitantly serve to recruit *Burkholderiales* cohorts with high sequence similarity to a nitrite-reducing isolate. This scenario may explain the nearly exclusive prevalence of the *Burkholderiales* order in Betaproteobacteria sequences across all incubation conditions in our study (**Table 2**). Very recently, the presence of low abundance *Arcobacter* spp. in 43-day mild steel incubations, coinciding with FeOB decline, was reported in West Boothbay Harbor, Maine (McBeth and Emerson, 2016). Lastly, *Arcobacter* spp. were reported as predominant in enrichments of Port of Los Angeles sediments in an electrochemical sulfide oxidation study (Li and Nealson, 2015), further associating this genus in MIC/ALWC activity near our study site.

### Implications for MIC/ALWC Near a Globally Important Port Complex

A full review of the complex ecological mechanisms responsible for marine MIC is beyond the scope of this work; however, this process may be briefly summarized as the microbiological influence of electrochemical or physical states at the interface of a metal surface and an aqueous layer by an exopolysaccharide-hosted, environmentally responsive and biologically dynamic biofilm (Von Wolzogen Kühr and van der Vlugt, 1934; Beech and Sunner, 2004; Beech and Campbell, 2008; Enning et al., 2012; Enning and Garrelfs, 2014). In this study, we aimed to assess the microbiological-chemical-physical interplay associated with ALWC, a phenomenon known to induce severe corrosion of steel pilings (Gubner and Beech, 1999).

Iron corrosion in anaerobic environments is understood to occur via two mechanisms: (1) an indirect mechanism where a chemical reaction occurs with hydrogen sulfide (Fe + H_2_S → FeS + H_2_); and (2) a direct mechanism where cathodic hydrogen reacts with iron directly (4Fe + SO_4_^2-^ + 4H_2_O → FeS + 3Fe^2+^ + 8OH^-^) (Dihn et al., 2004; Kakooei et al., 2012). *Desulfovibrio* spp., dominant in NE- and AF-FLOCS substrates (**Table 2**), are known to employ the direct mechanism for iron corrosion (Von Wolzogen Kühr and van der Vlugt, 1934; Hamilton, 2003; Dihn et al., 2004). This mechanism may facilitate the presence of reduced iron in an increasingly anoxic environment that, due to the concomitant reduction of sulfate to sulfide by SRB, may result in a lower pH at the metal-biofilm interface. A statistically significant decrease in pH beneath corrosion products of ALWC sites was previously documented (Gubner and Beech, 1999) and potentially explains the presence of the *Burkholderiales* order, with acidophilic Fe-oxidizing members (Fukushima et al., 2015), and *Desulfobacter* spp., capable of sulfate reduction coupled to metallic iron oxidation (Dihn et al., 2004), in AF-FLOCS substrates (**Figure 7**).

Sulfide oxidizing bacteria have been reported in statistically higher proportions at ALWC sites relative to standard MIC (Gubner and Beech, 1999). In the current study, relatively higher proportions of Epsilonproteobacteria sequences were recovered from NE-FLOCS (**Figure 6**) corroborating a potential link between eutrophic conditions and ALWC microbial community profiles. The majority of these sequences are classified as *Arcobacter* spp., a genus recently enriched from electrochemical sulfide oxidation studies of Port of Los Angeles sediment (Li and Nealson, 2015). We propose that this genus may play a role in the global phenomenon of ALWC and suggest further investigation into the microbiological-electrochemical role played by *Arcobacter* spp. in the potential establishment of a localized sulfur cycle likely responsible for ALWC.

Long-term incubations under nutritional enhancement, intended to simulate high iron and nutrient conditions associated with ALWC, revealed different microbial communities relative to non-nutritionally enhanced deployments (**Figure 8**). The high prevalence of Delta and Epsilonproteobacteria, specifically the *Desulfovibrio* and *Arcobacter* genera (**Figure 6**; **Table 2**), at end of a 6-month nutritionally enhanced enrichment (NE-FLOCS), relative to a non-nutritionally enhanced (AF-FLOCS) system, suggests that the environmental conditions associated with ALWC, result in: (i) scant evidence of Zetaproteobacteria-like “twisted stalk” structures such as those observed during short-term incubations, (ii) anoxic conditions, (iii) microbial communities capable of Fe and S cycling, and (iv) a disproportionately high amount of *Arcobacter* spp.- a genus indirectly (De Gusseme et al., 2009) and directly (Roalkvam et al., 2015) implicated in MIC. Our 2-month incubation study provides microscopic evidence of colonization by stalk-forming microorganisms that are hitherto only represented by Zetaproteobacteria in the marine environment. This is consistent with previous studies that have shown early colonization of mild steel by Zetaproteobacteria (Dang et al., 2011; McBeth and Emerson, 2016). It is likely that FeOB communities colonized our NE-FLOCS experiment at earlier times and seceded to other communities at later times under nutritional enrichment and associated redox (oxic/anoxic) transition.

## Conclusion

We conclude that surface pelagic microbial communities near the Port of Los Angeles exhibit a complex biological-chemical-physical response to the combined effects of iron and nutritional enhancement simulating ALWC. Our results indicate a successional biological response at each step of ferruginous substrate colonization; a process beginning with the proliferation of neutrophilic microaerophilic FeOB-like communities on mild steel substrates, followed by microbial communities comprised of S and Fe cycling Delta- and Epsilonproteobacteria, particularly under nutrient rich conditions. Our results also indicate that FeOOH products of neutrophilic FeOB metabolism precipitated under oxic conditions may, under anoxic conditions, serve as Fe(III) reduction sites. We propose that sulfide, the metabolic by-product of SRB, ultimately leads to the recruitment of SOB (*Arcobacter* spp., specifically) as the final step in a 3-tier [FeOB (*Mariprofundus ferrooxidans*-like) → SRB (*Desulfovibrio* spp.)/FRB (*Ferrimonas* spp.) → SOB (*Arcobacter* spp.)] ecological recruitment strategy for ferruginous substrate microbial colonization under eutrophic conditions, a process with implications for the enhanced MIC state known as ALWC near a globally important port.

## Author Contributions

GR, CH, and KE were responsible for the inception of this project. GR and CH were responsible for deployment and collection of the experiment, laboratory analyses, and data interpretation; ML contributed sequence data analysis and interpretation; RL contributed to deployment, recovery, and laboratory analyses; RB contributed to sample collection, experimental design and data interpretation; AG contributed to recovery, SEM analyses and data interpretation; BT mentored CH in synchrotron data analysis; and CW contributed major and minor trace element measurements. GR and BO wrote the paper with input from all authors.

## Conflict of Interest Statement

The authors declare that the research was conducted in the absence of any commercial or financial relationships that could be construed as a potential conflict of interest.

## Footnotes

1 www.Onlinemetals.com

2 support.illumina.com

3 http://unicorn.mcmaster.ca/aXis2000.html
